# Heterologous Expression of Bacteriocins from the Metagenome Mining of Cotija Cheese

**DOI:** 10.1007/s12602-025-10483-9

**Published:** 2025-02-26

**Authors:** Alfredo Esquivel-López, Diana Rocha-Mendoza, Carlos Eduardo Serrano-Maldonado, Alejandra Escobar-Zepeda, Maricarmen Quirasco

**Affiliations:** 1 Departamento de Alimentos y Biotecnología, Facultad de Química, Universidad Nacional Autónoma de México, Ciudad Universitaria, 04510 Mexico City, Mexico; 2 Mexico City, Mexico; 3 European Molecular Biology Laboratory, European Bioinformatics Institute (EMBL-EBI), Wellcome Genome Campus, Hinxton, Cambridge, UK

**Keywords:** Heterologous expression, Class IId bacteriocin, Metagenomic mining, Cotija cheese, Antilisterial activity

## Abstract

Bacteriocins are a heterologous group of ribosomal peptides with antibacterial activity. They are of interest to the pharmaceutical and food industries due to their potential to fight antibiotic-resistant pathogens and improve microbial food safety, respectively. Metagenomic data mining for antibacterial activity is valuable for the information it provides from unstudied genomic sequences. Furthermore, the higher biosynthetic yield obtained by the heterologous expression of putative bacteriocins allows their subsequent purification and characterization. This work aimed to express antilisterial bacteriocins in *Escherichia coli* after obtaining their gene sequences by in silico mining the bacterial metagenome of Cotija cheese. This artisanal Mexican cheese is manufactured with unpasteurized milk and ripens for at least 3 months. Analyzing the Cotija cheese bacterial shotgun metagenome allowed us to select two sequences (QC1 and QC2) encoding novel Class IId bacteriocins belonging to the lactococcin family. These genes were expressed as (His)6-fusion proteins in *E. coli* BL21 (DE3) and showed high antimicrobial activity against *Listeria monocytogenes*, with a minimum inhibitory concentration of 78 µg/mL. QC1 and QC2 were tested against several pathogenic bacteria and showed activity exclusively against *L. monocytogenes*. QC2 has a novel sequence that showed no matches against the UniProt database. It was purified by Ni^2+^ affinity chromatography and retained its activity after heating at 70 °C for 30 min. As the sequences were obtained by genomic mining on a fermented food metagenome, QC1 and QC2 have potential applications as sanitizers in industrial food facilities where *L. monocytogenes* contamination is the most prevalent.

## Introduction

Up-to-date microbial food safety remains challenging because products can be contaminated with microorganisms during processing and storage. The nutritional composition of food is diverse and provides an ideal environment for the proliferation of pathogens such as *Listeria monocytogenes*, *Escherichia coli*, *Staphylococcus aureus*, *Vibrio cholerae*, *Salmonella enterica*, and *Bacillus cereus*, among others [1, 2]. Food contaminated with these bacteria poses a significant human health risk and entails economic losses [3]. In addition, the global prevalence of antibiotic-resistant pathogens is a major issue, and the search for new alternative chemicals with antimicrobial activity that could be used in food is an area of current interest [4]. Microorganisms are an important source of antimicrobial compounds, including a large family of ribosome-synthesized peptides, known as bacteriocins, which exhibit antibacterial activity against closely related strains [5]. In the food area, bacteriocins from lactic acid bacteria (LAB) are the most studied because they are classified as generally recognized as safe (GRAS); therefore, unlike antibiotics, they can be used in food products [6]. Generally, the genes needed for bacteriocin biosynthesis and secretion share a genomic context in clusters encoded in plasmids, chromosomes, or transposons, which include bacteriocin, resistance, processing, and secretion genes [5]. Bacteriocin production is common in bacteria and is apparently found in all genera; they are rather diverse, which has led to different classifications [7]. Cotter et al. [8] classified bacteriocins into three main groups for Gram-positive bacteria, including LAB: Class I, lantibiotics; Class II, non-lanthionine-containing bacteriocins; and Class III, bacteriolysins, which are lytic enzymes. Class II includes most bacteriocins, which are small (< 10 kDA), heat-resistant, non-lanthionine-containing peptides that, unlike lantibiotics, do not undergo extensive post-translational modifications [8]. Class II can be further divided into four categories: Class IIa consists of pediocin-like peptides with a conserved “YGVGN” motif at the N-terminal and a disulfide bridge formed by two cysteines at the C-terminal. Class IIb are two-peptide bacteriocins that require both peptides to work synergistically to be fully active. Class IIc is a family of bacteriocins with a circular peptide backbone arising from the covalent linkage of their N- and C-terminal residues. Class IId comprises the remaining bacteriocins that do not fit the above categories. This miscellaneous group includes non-pediocin single linear peptides, leaderless peptides, and multi-peptide bacteriocins [9, 10]. Additionally, Class IIa and some Class IId bacteriocins are active against *L. monocytogenes* [11, 12].

Listeriosis is a food-borne disease of concern, being the third leading cause of food-poisoning deaths worldwide. In the USA, there are 1600 cases of food-borne illnesses per year, with a 16% mortality rate [13]. According to its severity, listeriosis can be invasive or non-invasive. Both types of illness are of concern. However, invasive listeriosis is riskier due to its high mortality rate (20–30%) in populations with weakened immune systems, i.e., infants, pregnant women, older adults, and patients with organ transplants, cancer, and AIDS [14]. *L. monocytogenes* can survive in various environments, including low temperatures and high salt concentrations, and can be found in various foods [15].

In the food industry, bacteriocins have a high potential for application as a preservative for different products, alone or combined with another preservation method. Different ways of using bacteriocins have been explored, such as the direct application of the pure compound on food and the inclusion of LAB in the food matrix or their use in food package films [16]. In recent years, genomic mining has become a powerful tool for identifying gene sequences encoding putative bacteriocins that, under the study conditions, are expressed at low concentrations or not expressed at all. Besides, metagenome mining allows searching for genes of interest in the total microbial community without a cultivation step.

Cotija cheese is a Mexican artisanal food product made from raw cow’s milk that ripens naturally. It holds great historical and cultural significance in Mexico, and it is also of commercial importance. It is well known that the consumption of unpasteurized milk poses a significant human health risk due to the pathogens it may contain; however, it has been shown that Cotija cheese does not represent a risk to consumers [17, 18]. We analyzed the shotgun metagenome of Cotija cheese, and the results revealed that the bacterial community is composed mainly of three dominant genera, namely *Lactiplantibacillus*, *Leuconostoc*, and *Weissella*, in addition to more than 500 non-dominant genera belonging to 31 phyla of bacteria and archaea. Genes from pathogens such as *Salmonella*, *L. monocytogenes*, *Brucella*, ETEC, and EHEC *E. coli* or *Mycobacterium* were not found [19]. The analysis of the whole-metagenome shotgun allowed us to search for taxonomical and functional information, e.g., genes encoding bacteriocins. Escobar et al. reported several contigs that contained genes encoding bacteriocins and bacteriocin immunity genes [19]. In this work, we continued genome mining for bacteriocin-encoding genes in the Cotija cheese shotgun metagenome, searching for non-studied peptides with antibacterial activity. Their functionality was assessed by expressing those sequences in *E. coli*, and their bacterial inhibition activity was qualitatively characterized.

## Materials and Methods

### In Silico Analysis for Putative Bacteriocin Sequences

Genome mining was performed on the publicly available metagenomic data from Cotija cheese at the Sequence Read Archive (SRA) site with accession number PRJNA286900. The genomic context of the genes of interest in the shotgun metagenomic assembly of the Cotija cheese was visualized using the SnapGene® software v7.2.1. Bacteriocin-encoding sequences were predicted using BAGEL4 v1.2 [20], and their functional annotation was confirmed using the BLASTp suite in NCBI [21] versus the UniProtKB database (release 2023_04). We selected two genes for heterologous expression according to the following criteria: annotation for Class II bacteriocin, the presence of a contiguous immunity gene, and the absence of any report from previous studies. These two bacteriocins were called QC1 and QC2.

The bacteriocin-encoding genes were translated in silico using the Expasy Translate Tool v3.0 [22], and amino acid sequences were used to find similar bacteriocins through BLAST (Basic Local Alignment Search Tool) with the target database UniProtKB reference proteomes + Swiss-Prot on the UniProt website accessed in December 2023. The results were limited to an *E*-value threshold of 0.001. To place our bacteriocin sequences within a reference tree, we retrieved the sequences sharing the “Protein names” of our BLAST hits “Lactococcin family bacteriocin” and “Bacteriocin lactococcin-A” from the UniprotKB + TrEMBL databases. From this list, only protein sequences with a length within approximately 20 amino acids relative to our queries were included in the upstream analysis. A total of 204 sequences that included QC1 and QC2 were dereplicated (100% identity, 100% coverage) using CD-HIT v4.8.1 [23]. The remaining 162 unique sequences were used to generate a multi-sequence alignment using Mafft v7.52 [24] with the default options. A maximum likelihood phylogenetic tree was built following the tutorial described by Vinuesa [25] to select the best model for our dataset based on the lnL score and the Akaike information criterion (AIC) estimation using PhyML v3.3 [26]. The final tree was generated with PhyML using the following parameters: `-m VT -f e -c 4 -a e -s BEST –rand_start –n_rand_starts 5 -o tlr `. The resulting tree was rooted at the midpoint; non-informative clades made of TrEMBL sequences only and sequences deleted in UniProt release 2024_01 were pruned in iTOL v6.8.1 [27]. The multisequence alignment of relevant sequences was edited for better visualization in Jalview v2.11.3.2 [28]. The tertiary structure of QC1 and QC2 was modeled using AlphaFold 3 [29].

### Bacteriocin Gene Cloning

The nucleotide sequences of QC1 and QC2 were synthesized by Epoch Life Science, Inc. (TX, USA) and deposited in GenBank with accession numbers PP919095 and PP919096, respectively. The QC2 leader sequence was predicted using SignalP v4.1. [30]. QC1 and QC2 were flanked at the 5′ and 3′ ends with sites for the restriction enzymes *Bam*HI and *Hind*III, respectively, and inserted into pUC18 vectors (100 ng/µL). Afterward, *E. coli* DH5α competent cells were transformed with each pUC18 vector using the thermal-shock technique [31]. Randomly selected ampicillin-resistant clones were purified individually using the GeneJET Plasmid Miniprep kit (Thermo Fisher Scientific, MA, USA).

### Subcloning Bacteriocin Genes

QC2 and QC1 were removed from the pUC18 vector using the enzymes *Bam*HI (Promega, WI, USA) and *Hind*III (Thermo Fisher Scientific, MA, USA) under the following conditions: 1 µg of plasmid, 1 µL of *Bam*HI, 2 µL of *Hind*III, and 2 µL of buffer R; the reaction mixture was brought to 20 µL with nuclease-free water. Then, this mixture was incubated at 37 °C for 2 h, followed by 20 min at 80 °C for enzyme inactivation. The digestion mixture was analyzed by electrophoresis in a 1% (w/v) agarose gel to verify the size of the inserted fragments. The QC1 and QC2 inserts were excised from the gel and purified using the GeneJET Gel Extraction Kit (Thermo Fisher Scientific, MA, USA). The purified inserts were then subcloned in the plasmid pET28a( +) (GenScript, NJ, USA), which has a kanamycin resistance gene and a His-tag at the multiple cloning site. The ligation conditions were the following: 50 ng of plasmid, 1 U of ligase, 2 µL of 10X ligase buffer, and 150 ng of insert. The mixture was brought to a final volume of 10 µL with nuclease-free water and incubated at 22 °C for 30 min for pET28a( +)-QC1 and for 5 h for pET28a( +)-QC2. The reaction was heat-inactivated at 70 °C for 5 min. Then, each ligation product was used to transform *E. coli* BL21(DE3) (Thermo Fisher Scientific, MA, USA) by thermal shock. Since the plasmid pET28a( +) has the T7 promoter and terminator sequences flanking the multiple cloning sites, the insertion of each construct was confirmed by analyzing some kanamycin-resistant clones with PCR using universal T7 primers [31]. To corroborate the sequence, the plasmids pET28a( +)-QC1 and pET28a( +)-QC2 were extracted from positive clones using the GeneJET Plasmid Miniprep kit (Thermo Fisher Scientific, MA, USA) and then sequenced by Macrogen, Inc. (Seoul, Korea).

### Induction of Transgenic Bacteriocins and Obtention of the Cytosolic Fraction

A five-milliliter pre-inoculum of the clones containing pET28a( +)-QC1 and pET28a( +)-QC2 was prepared in Luria–Bertani (LB) broth (Condalab, Madrid, Spain) with kanamycin (30 µg/mL) (LBK) at 37 °C and 250 rpm for 12 h. A 500 µL aliquot of the pre-inoculum was used to inoculate 50 mL of LBK broth in 250-mL flasks, which was then incubated at 37 °C and 250 rpm until it reached an optical density (OD_600nm_) of 0.5 to 1.0. Then, the inductor, isopropyl-β-D-1-thiogalactopyranoside (IPTG, Sigma-Aldrich, MO, USA), was added to a final concentration of 0.4 mM, and the flasks were incubated at 20 °C and 250 rpm for 5 h. The same procedure was performed to cultivate native *E. coli* BL21(DE3) and *E. coli* BL21(DE3) with empty pET28a( +) plasmids, which were used as controls.

After incubation, cultures were centrifuged at 4 °C and 10,000* g* for 15 min (Biofuga PrimoR Heraeus, Fisher Scientific, MA, USA); the supernatant was discarded, and the cell pellet was resuspended in a 10 mM Tris–HCl solution, pH 7.5, until reaching an OD_600nm_ of 2 to 2.5. The suspension was sonicated (F550 Sonic Dismembrator, Fisher Scientific, MA, USA) at 4 °C and 22 kHz with 20 s pulses alternating with 20 s rest for 7 min. After sonication, cells were centrifuged at 35,000* g* (Beckman Coulter J2-MC, CA, USA) and 4 °C for 20 min. Then, the supernatant was filtered through a 0.22-µm membrane (Merck-Millipore, Darmstadt, Germany). This preparation is hereafter referred to as the “cytosolic fraction.” Subsequently, an aliquot was freeze-dried, and the powder was suspended in a 10 mM Tris–HCl solution, pH 7.5, to a concentration of 5 mg/mL; this preparation is referred to as the “concentrated cytosolic fraction.” Protein concentration was measured using the Bradford protein assay (Bio-Rad, CA, USA) with a bovine serum albumin curve as a protein standard.

### Antimicrobial Activity Using the Agar Diffusion Test

The bacteriocins encoded in QC1 and QC2 were tested against the following bacteria: *L. monocytogenes* (CFQ-B-103), *Listeria innocua* (CFQ-B-232), *S. aureus* (ATCC 6538), *Enterococcus faecalis* (ATCC 29212), *B. cereus* (CFQ-B-230), *Streptococcus pyogenes* (CFQ-B-218), *S. enterica* Typhimurium (ATCC 14028), *Pseudomonas aeruginosa* (ATCC 27853), *Yersinia enterocolitica* (CFQ-B-231), and *E. coli* DH5α. All strains are deposited in the Culture Collection of the School of Chemistry (CFQ) at the *Universidad Nacional Autónoma de México* (UNAM) (WDCM No. 100). A pre-inoculum of the target microorganism was prepared in 5 mL of BHI broth (Brain Heart Infusion, Becton, Dickinson and Co., NJ, USA), which was incubated overnight at 37 °C. Then, 20 mL of BHI broth with 1.0% agar was poured into Petri dishes; later, 800 µL of a dilution (in a 0.85% [w/v] NaCl solution) of the pre-inoculum (OD_600nm_ = 0.4) was added to 9.2 mL of BHI broth with 0.8% (w/v) of agar. This 10 mL mixture was poured on top of the first layer of agar and left to solidify. Afterward, wells were made on the upper agar, and 200 µL of the cytosolic fraction to be evaluated was added to each. The Petri dishes were incubated at 37 °C for 12 h. Nisaplin® (Sigma-Aldrich, MO, USA) was used as a positive control, and native *E. coli* BL21(DE3) and *E. coli* BL21(DE3) with empty pET28a( +) plasmids were used as negative controls. This procedure was carried out with the cytosolic and concentrated cytosolic fractions. After incubation, Petri dishes were examined for the presence or absence of an inhibition halo around each well.

### Zymography Against *Listeria monocytogenes*

The mass of antibacterial proteins was estimated by zymography using Tris-Tricine-SDS-PAGE with *L. monocytogenes* as the substrate, as described by [32]. The translucent bands observed in the gel after destaining indicate lytic activity against the indicator microorganism. The molecular masses were determined using the Polypeptide SDS-PAGE Standard (Bio-Rad Laboratories Inc., CA, USA).

### Western Blot

To corroborate that the antibacterial protein corresponds to the expression of the bacteriocin transgene, a Tris-Tricine-SDS-PAGE was performed, followed by His-Tag identification by western blot. The proteins separated by Tris-Tricine-SDS-PAGE were transferred to a polyvinylidene difluoride membrane (PVDF; Bio-Rad Laboratories Inc., CA, USA) at 20 V for 40 min using a Trans-Blot® SD Semi-Dry Transfer Cell (Bio-Rad Laboratories Inc., CA, USA) and a transfer buffer (25 mM Tris, 190 mM glycine and 10% [v/v] methanol). After the protein transfer, the protein standard lane on the membrane was cut off and stained with Ponceau Red (Sigma-Aldrich, MO, USA); the rest of the membrane was blocked using a 3% (w/v) skim milk solution (Difco, Thermo Fisher Scientific, MA, USA) in TBE buffer (Tris-10 mM HCl, 150 mM NaCl, pH 7.5) with 0.05% (v/v) Tween-20 under gentle shaking at room temperature for 1.5 h. The membrane was then washed three times, for 1 min each, with 20 mL of TBE + Tween-20 buffer; then, the anti-His-tag antibody coupled with alkaline phosphatase (Abcam, Cambridge, UK) diluted 1:2000 in 20 mL of the same buffer was added; it was left at room temperature for 1 h under gentle stirring. Finally, the membrane was washed twice with TBE + Tween-20 buffer; then, the NBT/BCIP revealing solution (Thermo Fisher Scientific, MA, USA), diluted 1:2 in distilled water, was added until purple bands were observed, showing the antibody-histidine tag.

### Minimum Inhibitory Concentration (MIC)

The minimum inhibitory concentration against *L. monocytogenes* was determined in duplicate following the method described by Andrews with some modifications, as described below [33]. *L. monocytogenes* was pre-cultured in 5 mL of LB broth (incubated at 37 °C for 16 h); then, a 1 × 10^−6^ dilution in fresh LB broth was prepared. On the other side, the concentrated cytosolic fractions of the clones containing pET28a( +)-QC1 and pET28a( +)-QC2, as well as native *E. coli* BL21(DE3) and *E. coli* BL21(DE3) with empty pET28a( +), were adjusted to 5 mg protein/mL. From each preparation, serial doubling dilutions were made, giving a total of ten different concentrations ranging from 5 to 0.01 mg/mL. A 30 µL aliquot of each dilution was mixed with 30 µL of the previously prepared dilution of *L. monocytogenes*, and the mixture was incubated at 37 °C for 24 h. After incubation, each mixture was homogenized again, and a 10 µL aliquot was poured into a Petri dish with LB agar (Condalab, Madrid, Spain). Once the samples were absorbed in the agar, plates were incubated at 37 °C for 24 h. The MIC was determined as the protein concentration at which no colony-forming units (CFUs) were observed. Nisaplin® (Sigma-Aldrich, MO, USA) was used as a positive control, and the starting protein concentration for the serial doubling dilutions was 200 µg protein/mL.

### Purification of QC2 Bacteriocin

His-tagged bacteriocin was purified by affinity chromatography using a silica resin with Ni^2+^ ions (Protino® Ni-TED, Thermo Fisher Scientific, MA, USA). Approximately 2 g of resin was used, following the supplier’s instructions. The pET28a( +)-QC2 cytosolic extract was obtained by sonication as described above using the denaturing Lysis-Equilibration-Wash buffer (LEW) (8 M urea, 2 M NaCl, 50 mM NaH_2_PO_4_, 10 mM imidazole, pH 8); this buffer was also used to equilibrate the resin. His-tagged bacteriocin was eluted using LEW buffer added with 250 mM imidazole. Afterward, dialysis was carried out using a Spectra/Por® membrane (RepliGen, MA, USA) with a cutoff of less than 1 kDa. The urea concentration was gradually eliminated using a buffer solution of 2 M NaCl, 50 mM NaH_2_PO_4_, 10 mM imidazole, pH 8, with different urea concentrations (4, 2, and 1 M), and a final buffer of 10 mM Tris–HCl, pH 7.5. Dialysis was carried out at 4 °C with moderate stirring, and the buffer solution was replaced every 4 h with decreasing urea concentrations. This sample was filtered through a 0.22-µm membrane (Merck-Millipore, Darmstadt, Germany), freeze-dried, and the powder was suspended in a sterile 10 mM Tris–HCl solution, pH 7.5. Finally, the sample was analyzed by SDS-PAGE and western blot, and its thermal stability was evaluated.

### Thermal Stability

The purified bacteriocin was aliquoted, and each volume was exposed to different temperatures (50, 70, and 90 °C) for 30 min. Subsequently, 50 µL of a diluted suspension (1:1000, using sterile 10 mM Tris–HCl, pH 7.5) of *L. monocytogenes* previously cultured in LB broth and incubated at 37 °C for 12 h was added to each 50 µL bacteriocin aliquot. The mixture was vigorously homogenized and incubated at 37 °C for 24 h. An *L. monocytogenes* growth control (using the 1:1000 dilution) and a sterility control (LB broth diluted 1:1 with the same Tris–HCl sterile buffer) were used. Finally, 10 µL of each mixture was poured on LB agar in duplicate and incubated at 37 °C for 18 h.

The newly determined nucleotide sequences of QC1 and QC2 are deposited in GenBank with accession numbers PP919095 and PP919096, respectively.

## Results

### In Silico Characterization of Metagenomic Bacteriocins

**Fig. 1:**
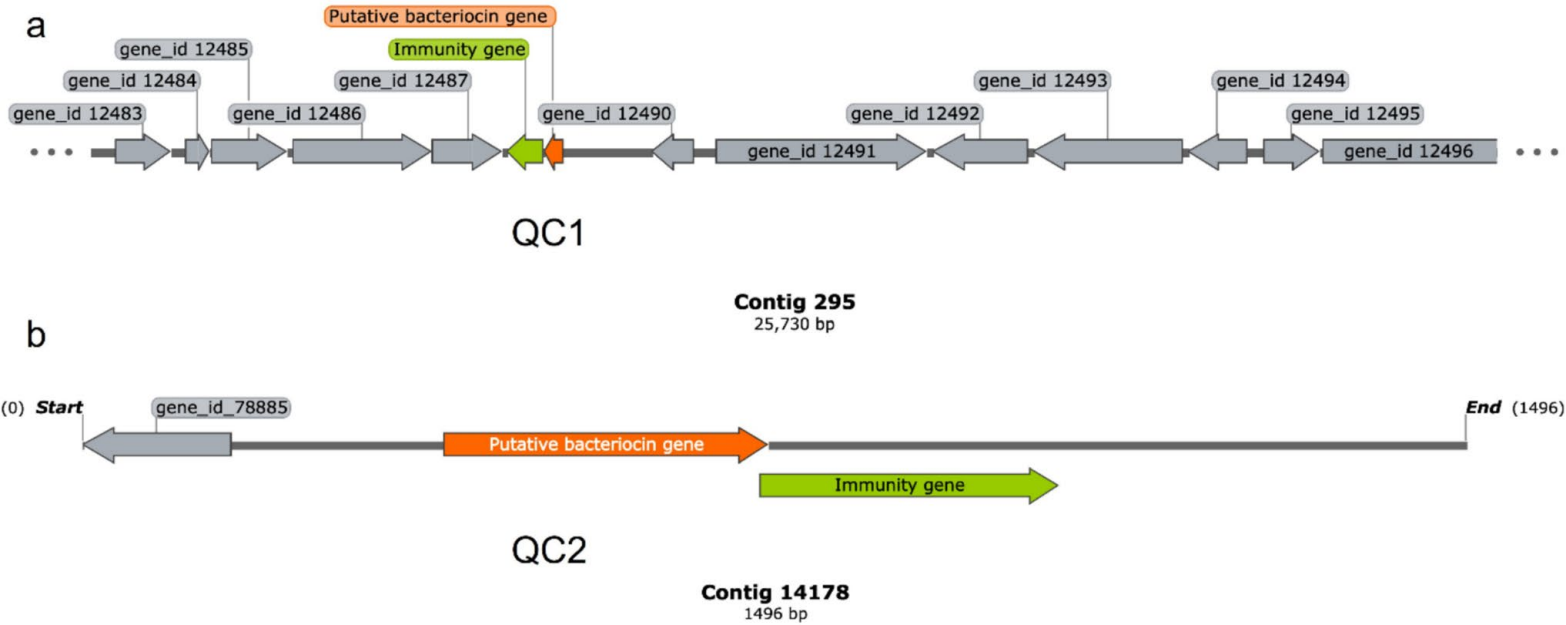
Genomic context of the genes of interest in the shotgun metagenomic assembly of the Cotija cheese visualized using SnapGene®. The bacteriocin structural gene is shown in orange and the immunity gene in green. **a** QC1 on contig 295 is composed of the immunity gene (303 bp) and the bacteriocin gene (165 bp). **b** QC2 on contig 14178 contains a bacteriocin gene (267 bp) and its immunity gene (321 bp). The numeral corresponds to the metagenomic assembly generated in [19] from the raw reads available on the PRJNA286900 SRA BioProject

The bioinformatics mining of the Cotija cheese bacterial shotgun metagenome identified 43 contigs with at least one bacteriocin or immunity gene [19]. Of these, 19 contigs were selected for analysis because they were annotated as containing a bacteriocin-encoding gene contiguous to an immunity one, suggesting they belong to the same biosynthetic gene cluster. Afterward, a BLAST analysis and a search in UniProtKB and Bagel4 were performed to obtain information about the putative bacteriocin gene (e.g., classification, target microorganism, or putative-producing bacteria). As a result, we found eight known bacteriocins: two enterocins P, one pediocin PA-1, one hiracin-JM79, one enterocin A, one enterocin NKR-5-3C, and one bacteriocin SRCAM 602, all of them identified as Class II bacteriocins by Bagel4, in addition to another sequence identified as bovicin 255. Five putative bacteriocin genes did not match any sequence in UniProtKB or NCBI-nr but were classified as Class II bacteriocins by Bagel. Finally, the last six sequences were annotated as hypothetical or uncharacterized proteins in NCBI-nr and UniProtKB, whereas Bagel identified them as belonging to the lactococcin family. With this information, we shortlisted two putative bacteriocin genes (named QC1 and QC2 in this work) that encode hypothetical bacteriocins and have a contiguous immunity gene. The genomic context of the selected genes in the metagenomic assembly is shown in Fig. 1.

**Fig. 2:**
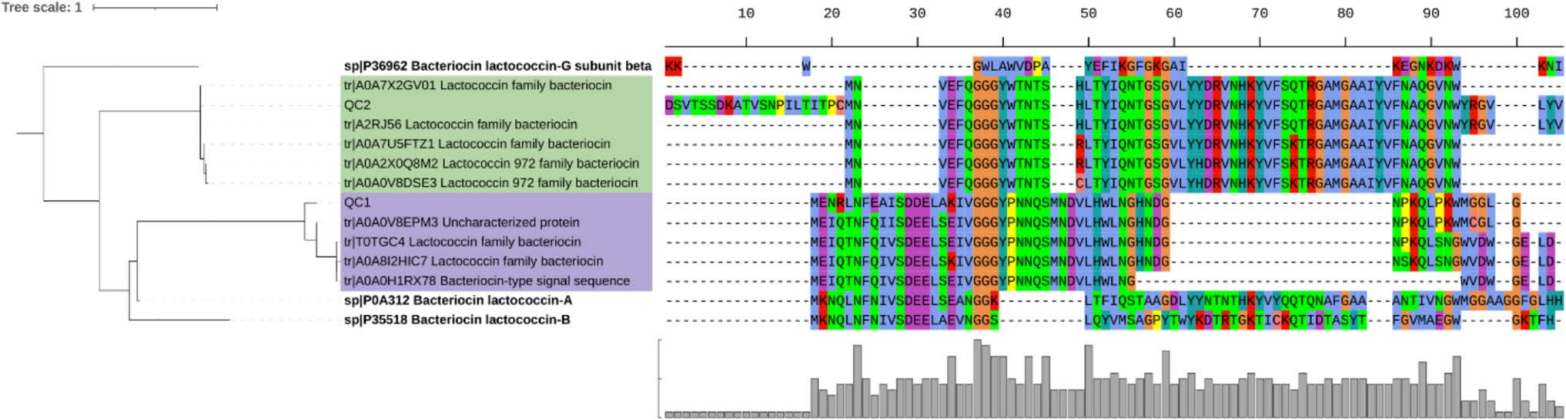
Maximum likelihood phylogenetic tree of protein sequences retrieved from the UniProt database based on sequence and annotation similarity to QC1 and QC2. Sequences related to clades QC1 and QC2 with annotation in the SwissProt database (sp prefix) are highlighted in bold. Sequences grouped with QC1 and QC2 belong to the TrEMBL database (tr prefix). The conservation graph is shown at the bottom of the multisequence alignment

An in silico translation and leader sequence prediction of the annotated gene QC2 in contig 14178 yielded a 116-residue peptide with a possible N-terminal region with a cleavage site before the D29 residue and not after the double-glycine, as is typical for Class II bacteriocins. Accordingly, we decided to clone the QC2 gene starting from the D29 codon, resulting in an 88-residue peptide, used for the amino acid alignment shown in Fig. 2. A subsequent analysis versus reference sequences revealed five sequences identical to QC1 in the TrEMBL database (accession numbers A0A0B8QXJ4, A0A2A5SQY3, A0A2A9I9W4, A0A6I2HHP4, A0A5D4G3W1). These proteins are annotated as glycosyltransferase, lactococcin bacteriocin, or bacteriocin. On the other hand, QC2 had no identical sequences in the database. To understand the novelty of QC1 and QC2 in the context of previously reported bacteriocins, we generated a maximum likelihood phylogenetic tree of protein sequences similar to our bacteriocins. The topology of the resulting tree revealed that QC1 and QC2 belong to two different clades made of sequences with a low characterization level in the TrEMBL database (Fig. 2). However, in both clades, the sequence name states that they belong to Lactococcin-family bacteriocins.

**Fig. 3:**
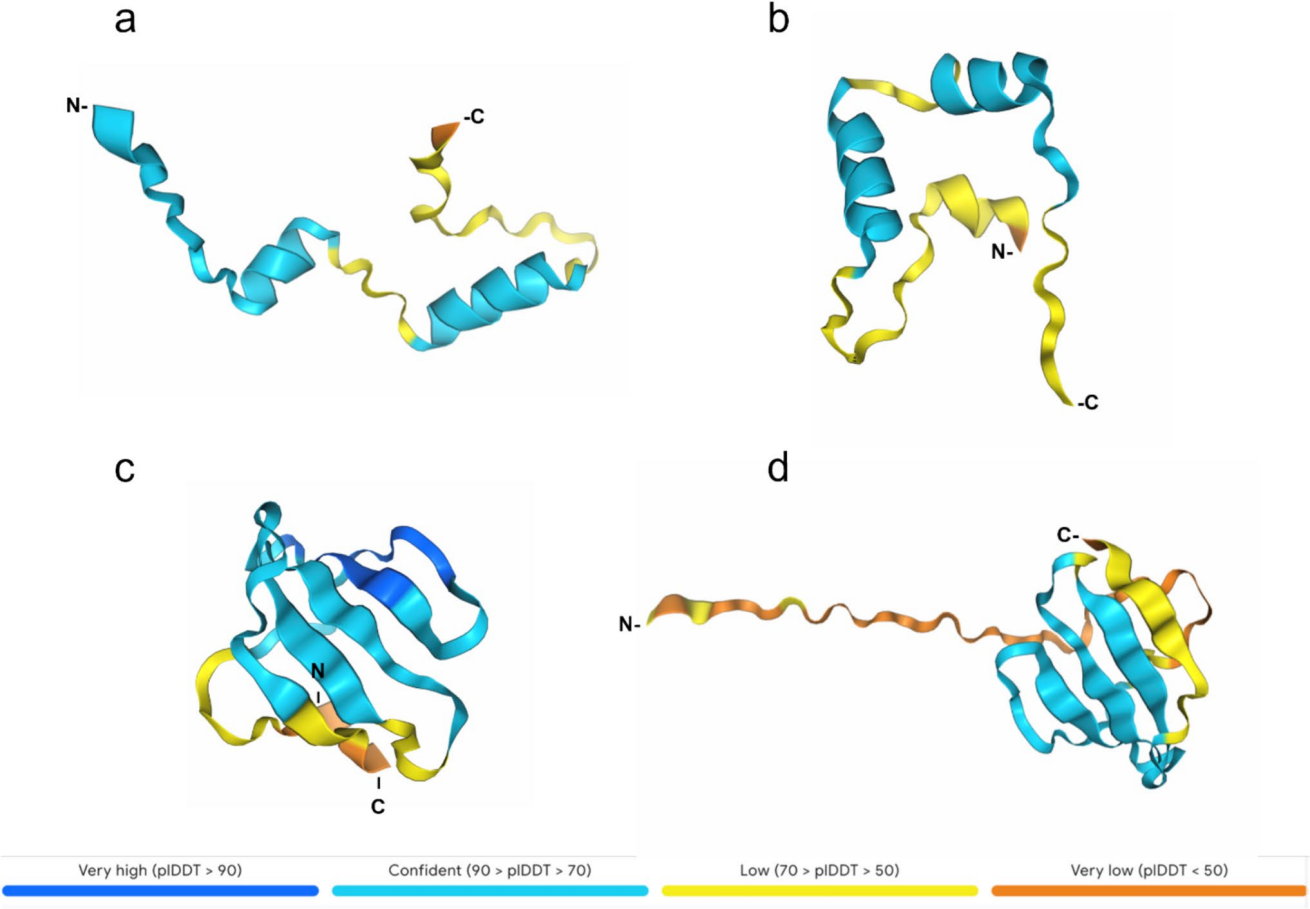
Comparison of the QC1 and QC2 models of tertiary structure versus the ones corresponding to the closest primary sequences depicted in the maximum likelihood phylogenetic tree (Fig. 2). **a** Model of tr|A0A0V8EPM3 with two α-helices. **b** Bacteriocin QC1 model showing two α-helices. **c** tr|A2RJ56 model showing three β-sheets. **d** Bacteriocin QC2 model with five antiparallel β-sheets and a unique N-terminal region. The color code corresponds to the confidence level established by AlphaFold 3

A multisequence alignment of proteins between the putative bacteriocins identified in this study and the reference sequences of lactococcin-family members revealed conserved regions in the sequences of interest. The bacteriocin QC1 has a high identity in most positions (51/55 amino acids) across the whole sequence to the uncharacterized protein A0A0V8EPM3 (Fig. 2). The latter is identical to a lactococcin predicted in *Lactococcus lactis* subsp. *cremoris* (strain MG1363) (tr|A2RJ39), a highly studied and deeply characterized model of lactococcal strains [34]. Although the bacteriocin QC1 has the highly conserved GG at position 20, which resembles the recognition site for the excision of the leader peptide, we decided to use the complete open reading frame (ORF). The 3D AlphaFold models generated for some of the sequences of clade QC1, available from Uniprot, showed a pattern of multiple α-helixes similar to those of the QC1 model. There are two well-studied Class IId bacteriocins, Lacticin Q and Lacticin Z, characterized by multiple α-helix domains [35, 36]. Although they do not appear in the same clade as QC1 in the phylogenetic tree, we observed the same pattern of multiple alpha helixes when comparing the bacteriocin QC1 model with the one reported for Lacticin Q [37]. Figure 3 shows the 3D modeling of the tertiary structure of the putative bacteriocins QC1 and QC2, along with the most similar ones according to the phylogenetic tree shown in Fig. 2.

On the other hand, QC2 has a unique portion of 21 amino acids at the amino-terminal. However, the rest of the sequence is identical to the lactococcin A2RJ56 predicted in *Lactococcus lactis* subsp. *cremoris* (strain MG1363) [34]. According to the likelihood phylogenetic tree (Fig. 2), QC2 is similar to bacteriocins belonging to the lactococcin 972 family. The three-dimensional QC2 model suggests that it differs from other bacteriocins reported to date (Fig. 3). Furthermore, neither QC1 nor QC2 can form disulfide bonds because they lack cysteine in their primary structure. There is still much to study about this polypeptide to describe its actual 3D structure and mechanism of action.

### Assessment of Antimicrobial Activity

**Fig. 4:**
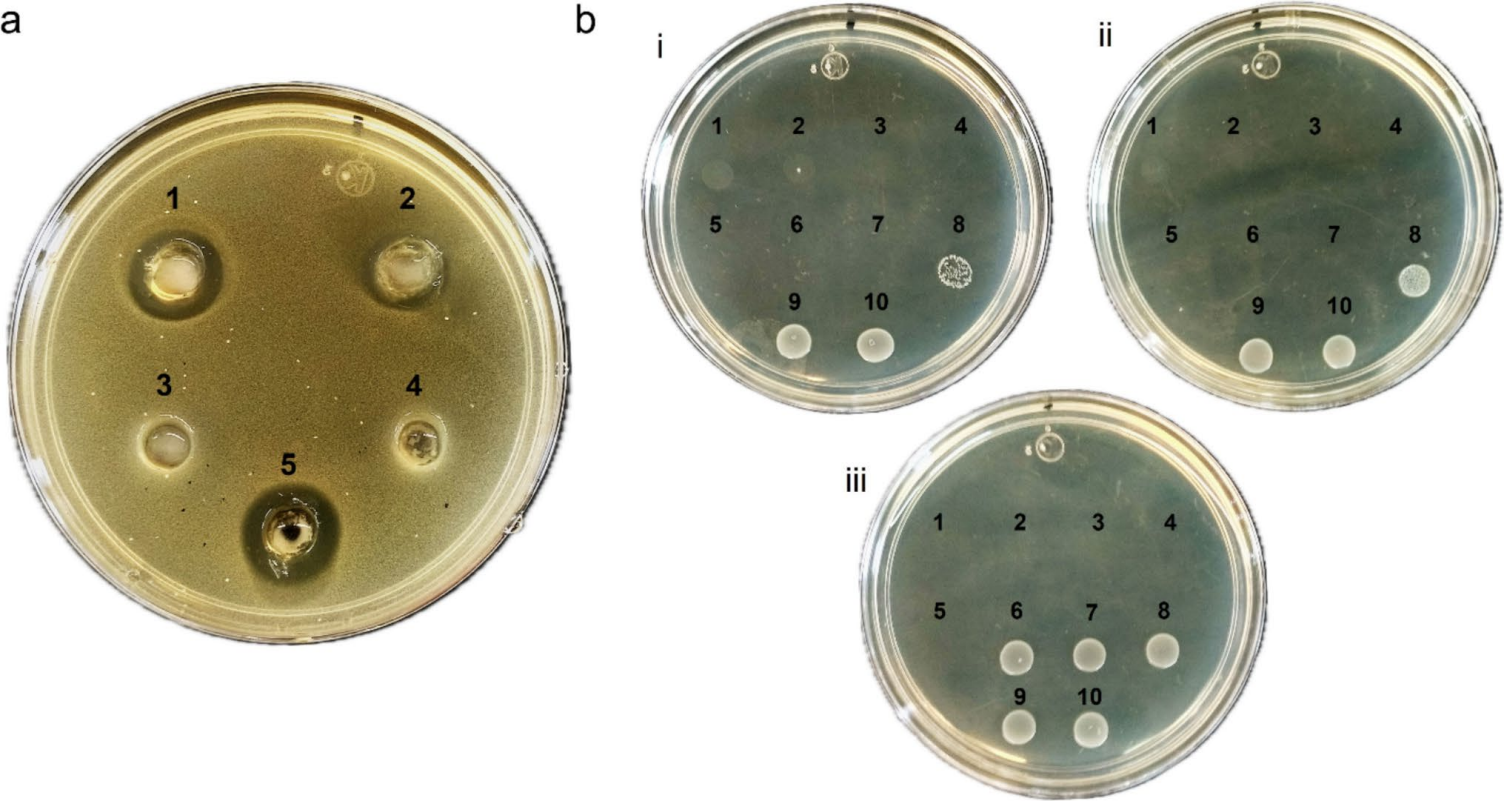
Antibacterial activity of QC bacteriocins. **a** Agar diffusion assay against *L. monocytogenes*: (1) QC1; (2) QC2 cytosolic extracts; (3) the empty pET28a( +) vector in *E.coli* BL21(DE3); (4) *E. coli* BL21(DE3); (5) Nisaplin® (Nisin), 10 mg/mL. **b** MIC against *L. monocytogenes*: (i) Bacteriocin QC1; (2) Bacteriocin QC2; (3) Nisaplin® (Nisin). In (i) and (ii), numerals 1 to 10 denote serial doubling protein dilutions ranging from 5 to 0.01 mg/mL. In (iii), 1 to 10 denote serial doubling protein dilutions ranging from 200 to 0.39 μg/mL

We decided to include the immunity protein in each construction considering that it might be useful to protect the producing strain from any deleterious effect of the recombinant bacteriocin. The in silico analyses showed that their amino acid sequences are not similar to any immunity protein reported previously. Thus, it would be interesting to assess each of them separately to better understand their mechanism of action. Consequently, the bacteriocins and their respective immunity protein genes were synthesized and cloned into *E. coli* BL21(DE3). The heterologous bacteriocins were expressed independently as cytosolic peptides, and their antimicrobial activity against *L. monocytogenes* was evaluated by agar diffusion assays. Figure 4a shows that the two heterologous bacteriocins (wells 1 and 2) showed antagonistic activity against *L. monocytogenes*. No antimicrobial activity was observed for *E. coli* BL21(DE3) extracts with and without the empty expression vector (negative controls; wells 3 and 4). The positive control, bacteriocin nisin (Nisaplin®), showed activity against *L. monocytogenes* (well 5). The minimum inhibitory concentration (MIC) against *L. monocytogenes* was assessed using the concentrated cytosolic extracts. A total of 10 different concentrations of the cytosolic extracts, ranging from 5 to 0.01 mg/mL, were evaluated in duplicate. The MIC was determined as the concentration at which no colony-forming units (CFU) were observed (Fig. 4b). Bacteriocins QC1 and QC2 inhibited the growth of *L. monocytogenes*, with an MIC of 78 µg/mL (see Fig. 4b(i) and (ii)); for reference, the MIC of the positive control was 12.5 µg/mL (Fig. 4b(iii)). The host strain *E. coli* extracts without the vector and with the empty vector did not show an inhibitory effect against *L. monocytogenes* in the MIC evaluation following the diffusion agar assay (Fig. 4a).

To assess the antibacterial activity of bacteriocins QC1 and QC2 against bacteria other than *L. monocytogenes*, agar diffusion assays were performed against the following microorganisms: *Staph. aureus*, *B. cereus*, *S. pyogenes*, *Salm. enterica* Typhimurium, *Ps. aeruginosa*, *Y. enterocolitica*, *Ent. faecalis, L. innocua*, and *E. coli* DH5α. No activity was observed against any of these strains.

### Characterization of the Novel Bacteriocin QC2

The pairwise alignment of sequences using BLAST versus comprehensive databases (NCBI-nr) revealed that the best match of QC2 corresponds to a putative bacteriocin from *Lactococcus lactis* with 97% similarity at the nucleotide level. We aimed to purify and characterize QC2 considering its novelty.

**Fig. 5:**
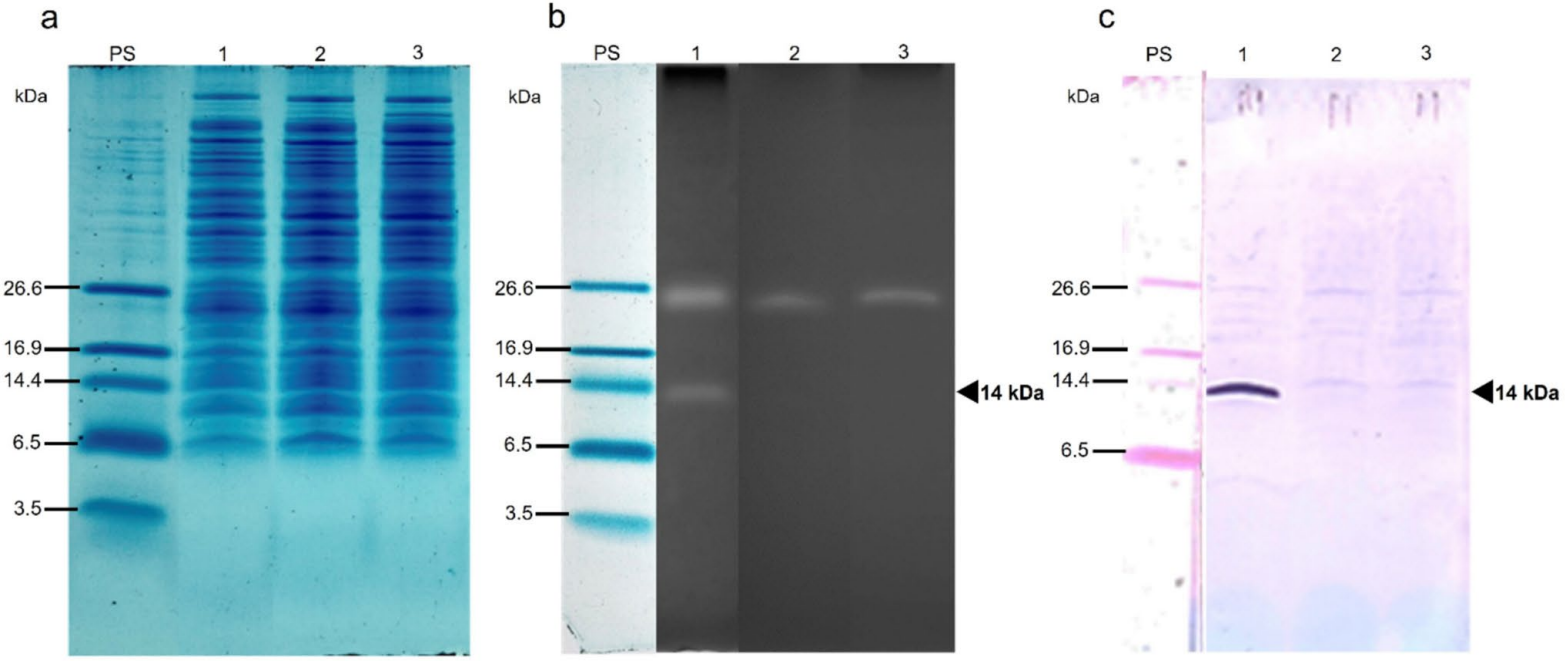
Characterization of cytosolic extracts. **a** Protein electrophoresis on SDS-PAGE. **b** Zymography against *L. monocytogenes.* **c** Western blot assay. Lanes: PS, protein standard; (1) QC2; (2) empty pET28a( +) vector in *E.coli* BL21(DE3); (3) *E. coli* BL21(DE3)

The cytosolic extracts of QC2, *E. coli* BL21(DE3), and *E. coli* BL21(DE3) with the empty vector (control) were analyzed by zymography using *L. monocytogenes* as the target strain. Additionally, an SDS-PAGE tris-tricine gel and a western blot anti-histidine tag were run with the same extract (Fig. 5). The zymogram allowed for the detection of translucent bands that represent lytic activity against the target microorganism. The expected QC2 molecular mass (~ 13.9 kDa), calculated by processing the leader peptide in the residue D29 with 38 additional amino acids belonging to the cloning vector, corresponds to a translucent band observed in the zymogram. However, another lytic band with an approximate weight of 26 kDa is also observed in the lane where the cytosolic extract of the host strain was analyzed; based on this finding, we presume it belongs to a native protein of *E. coli* BL21(DE3) (Fig. 5b). This activity was not reflected in the negative controls in the agar diffusion test or in the MIC assay (Fig. 4a). The western blot results showed that QC2 gives a signal around 14 kDa (Fig. 5c).

**Fig. 6:**
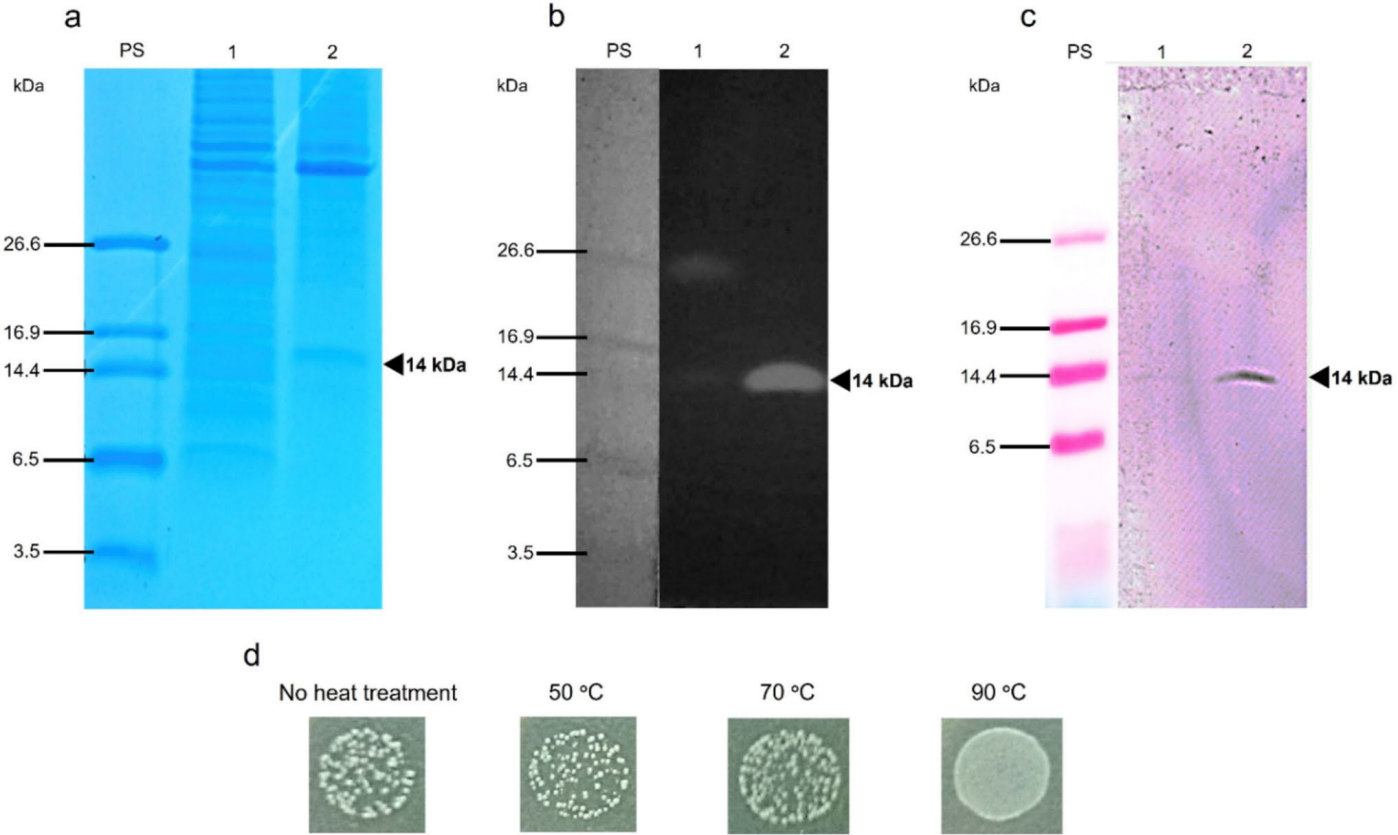
Protein QC2 after purification (5.6 µg/mL). **a** SDS-PAGE. **b** Zymogram versus *L. monocytogenes*. **c** Western Blot anti-His tag. **d** Thermal stability of purified QC2 (50 μL) assessed by its activity against *Listeria monocytogenes*

The bacteriocin QC2 was purified from the cytosolic extract by denaturing His-tag affinity chromatography. A fraction with a concentration of 5.6 µg/mL was further analyzed (Lane 2, Fig. 6a); after refolding, an intense lytic band was observed in the zymogram against *L. monocytogenes* (Lane 2, Fig. 6b). The purification of the recombinant bacteriocin allowed us to remove the presumptive *E. coli* lytic protein (26 kDa). Western blot confirmed that His-tagged bacteriocin had the expected molecular mass, i.e., approximately 14 kDa (Fig. 6c). It is noticeable that the His-tagged bacteriocin is active.

Thermal stability tests were carried out using purified bacteriocin QC2, exposing it to different temperatures (50 °C, 70 °C, and 90 °C) for 30 min; subsequently, its activity was evaluated as described in the determination of the MIC. We observed that the bacteriocin QC2 retains its activity after exposure to 50 °C and 70 °C for 30 min; its activity is lost after exposure to 90 °C for the same time (Fig. 6d).

## Discussion

Cotija cheese is made with unpasteurized milk; it ripens naturally for at least 3 months and poses no microbiological risk to consumers [17–19]. A wide variety of bacteria have been identified in the ripened product, where the predominant species are LAB, namely *Lactiplantibacillus plantarum*, *Leuconostoc mesenteroides*, and *Weissella paramesenteroides* [19]. The presence of LAB and the physicochemical changes during the ripening process (i.e., decrease in a_W_ and pH concomitant with increased acidity) could explain the absence of pathogenic bacteria such as *Salmonella*, *Brucella*, ETEC and EHEC *E. coli*, or *L. monocytogenes*. Additionally, molecules such as bacteriocins could contribute to the control of pathogenic bacteria due to their antibacterial activity. Since we have metagenomic information of the bacterial community in Cotija cheese obtained by a whole-metagenome shotgun, genomic mining was the next step in looking for novel bacteriocins without the need for bacterial culture. Furthermore, the high number of reported bacteriocin sequences allows the enrichment of the databases, which positively affects the prediction accuracy of tools [20].

The contigs of the Cotija cheese shotgun metagenome analyzed with BAGEL4 revealed that some of the identified bacteriocins were already characterized; four of them belong to the *Enterococcus* subdominant genus in the Cotija cheese bacterial shotgun metagenome and one to *Pediococcus* [19]. Additionally, we identified two DNA sequences encoding Class II bacteriocins, namely QC1 and QC2, whose peptides had not been previously studied and whose antimicrobial activity may be involved in the Cotija cheese ripening process. Many Class II bacteriocins are active against different Gram-positive bacteria, with a high specificity for *L. monocytogenes*, representing an interesting alternative to deal with this food-borne pathogen [16]. Considering that a bacteriocin gene and an immunity gene are usually part of a cluster, where the immunity gene encodes a protein that confers resistance to the producing strain, we used both genes in the constructions [5, 38].

The bacteriocin QC1 is a 73-amino acid antilisterial peptide. Its theoretical isoelectric point and net charge at pH 7.0 are 6.726 and − 0.35, respectively; its hydrophobic ratio is 32, with a calculated mass of 7,940 Da. The bacteriocin QC2 is an antilisterial protein composed of 126 amino acids; it is heat-stable, with a theoretical mass of 13,766 Da, and a hydrophobic ratio of 31%. Its calculated isoelectric point and net charge at pH 7.0 are 8.963 and + 3.399, respectively. Both bacteriocins lack the Tyr-Gly-Asn-Gly-Val-X-Cys-X-X-X-X-Cys-X-Val consensus sequence at their N-terminal region, characteristic of pediocin-like bacteriocins; they are not circular and do not act as a dipeptide. The above characteristics allow us to group QC1 and QC2 bacteriocins into Class IId [39–41], targeting *L. monocytogenes* according to their different structural characteristics. These bacteriocins have promising commercial potential since they can be readily synthesized without cleaving the leader peptide to make them active, facilitating their production [16].

The two α-helices predicted in the bacteriocin QC1 model suggest a structural similarity to the lacticins Q and Z (leaderless bacteriocins) produced by *L. lactis* QU 5 and *L. lactis* QU 14, respectively [16, 36, 41]. These homologous cationic peptides have 53 amino acids and a formylated methionine at the N-terminus and show high antimicrobial activity at nanomolar concentrations. In particular, lacticin Q shows a MIC of 750 nM against *L. innocua* ATCC 33090 [16, 35]. Of the pathogenic microorganisms tested, the bacteriocin QC1 is active only against *L. monocytogenes* (with a MIC of 78 µg/mL, equivalent to 9.82 μM); these findings contrast with observations reported for lacticins Q and Z, which are active against other Gram-positive bacteria [35, 41]. Yoneyama et al. [42] reported that lacticin Q binds rapidly to phospholipids in the bilayer membrane, forming toroidal pores (4.6–6.6 nm). This type of pore appears for a limited period, i.e., the time taken for lacticin Q to translocate from one side of the membrane to the other due to its helical structure [42]. The bacteriocin QC1 model suggests that it may interact similarly with the cell membrane due to several α-helixes in its structure and its hydrophobic ratio.

On the other hand, QC2 showed a close similarity with bacteriocins in the lactococcin 972 family. Lactococcin 972 (Lcn972) produced by *Lactococcus lactis* subsp. *lactis* IPLA972, is a 7.5-kDa 66-amino acid polypeptide with a sandwich anti-parallel β-sheet structure, functioning as a homodimer. It is not a hydrophobic peptide; therefore, its mechanism of action does not involve pore formation and leakage of cytoplasmic components. In contrast, it inhibits the growth of sensitive bacteria by disrupting septum formation in dividing cells by blocking the incorporation of the cell wall precursor N-acetylglucosamine [43, 44]. Although Lcn972 targets lipid II, it is not a lantibiotic [45]. Bacteriocin QC2 lacks the pediocin-like box in its amino acid sequence; accordingly, it does not belong to Class IIa. We suggest including it in Class IId, a highly diverse group that encompasses any peptide that does not fit into the other groups of Class II bacteriocins. Bacteriocins are expected to be highly diverse, considering their abundance in nature. It has been estimated that 99% of bacteria produce at least one bacteriocin [46], which implies abundant unknown bacteriocins produced by different bacteria. Bacteriocin QC2 differs from others in Class IId, but this study nonetheless demonstrated its activity against *L. monocytogenes*. A comparison of the activity between QC2 and other lactococcins in the 972 family is currently not possible due to the lack of information on the MIC of bacteriocins of this type; thus, further studies addressing the lactococcin 972 family are required. Given that its mechanism of inhibitory action is markedly different from that of other bacteriocins, this group is of particular interest. Regarding the DNA sequence encoding QC2 in the cheese shotgun metagenome, Escobar et al. [19] indicated that *Lactococcus lactis* is one of the non-dominant species present in Cotija cheese, suggesting that the QC2 sequence could belong to this LAB species.

Class II bacteriocins are known for their high thermal stability, largely due to their low mass and, in some cases, disulfide bonds that enhance molecule stability; however, there is scarce documented evidence regarding this property in Class IId bacteriocins. One example is Lcn972, which is thermosensitive and loses its structure and antimicrobial activity after 4 h at 25 °C and a low pH [43]. On the other hand, Li et al. [47] reported that the bacteriocin CAMT6 from *Enterococcus durans* YQ-6, also belonging to Class IId, is a 1.25-kDa peptide that showed 92% residual activity after heating at 100 °C for 15 min [47]. QC2 maintains full activity at 70 °C for 30 min at pH 7.5, and considering its mass and the lack of disulfide bonds, it could have potential use in products subjected to mild thermal processing.

## Conclusion

Our research group has successfully produced two novel antilisterial Class IId bacteriocins in the laboratory through genome mining in the metagenomic data of an artisanal ripened cheese made from raw cow milk. One advantage of their narrow activity spectrum is that they can be used against the specific target pathogen without altering the commensal population in a fermented product, which is an advantageous property in food production. Their MIC against *L. monocytogenes* makes them competitive with other bacteriocins from lactic acid bacteria. However, novel molecules must undergo a thorough characterization before commercialization, including cytotoxicity tests, mechanism of action, antibacterial activity spectrum, release mode, stability, and other factors. Given the ongoing public health concern of food-borne listeriosis, particularly associated with dairy products, we propose the application of the novel bacteriocins QC1 and QC2 as antilisterial sanitizing agents on surfaces of milking equipment or food processing facilities. It should be noted that safety testing before approval will depend on the specific sanitizing product, its intended application, and the legislation of each country. Considering the proposed use of these products in the food industry, the results of this research may serve as a foundation for their subsequent expression in a GRAS microorganism, such as *Lactococcus lactis*.

## Data Availability

The metagenomic data from Cotija cheese is publicly available at the SRA with accession number PRJNA286900. QC1 and QC2 sequences are available in GenBank with accession numbers PP919095 and PP919096, respectively.
